# Computational systems biology: integration of sequence, structure, network, and dynamics

**DOI:** 10.1186/1752-0509-5-S1-S1

**Published:** 2011-06-20

**Authors:** Yong Wang, Xiang-Sun Zhang, Luonan Chen

**Affiliations:** 1 Academy of Mathematics and Systems Science, Chinese Academy of Sciences, Beijing 100190, China; 2 Key Laboratory of Systems Biology, Shanghai Institutes for Biological Sciences, Chinese Academy of Sciences, Shanghai 200031, China

## Abstract

A report of the 4nd International Conference on Computational Systems Biology, 9-11 September 2010, Suzhou, China.

## Background

Computational systems biology, a term proposed in 2002, focuses on the system-level analysis on biological data using computational methods [1]. In essence, computational systems biology is a marriage between systems biology and computational biology and covers many different aspects of this energetic field. Comparing to this new term, systems biology emphasizes the integration of experimental and computational research to understand the complex biological systems, while computational biology highlights the pragmatic modelling and theoretical exploration to address critical biological questions [2-5].

Computational systems biology represents an interdisciplinary research style by bridging methodologies and biological problems and further strengthening their feedback interaction at a system-wide level. Four years ago, we launched an international symposium on Optimization and Systems Biology, which is also interdisciplinary by its nature, and aims to bridging opportunities between optimization methodology and systems biology problem [6]. We are glad to see that more and more manuscripts on this area have been submitted to the meeting, and many of them not only present the great potential to solve specific biological problems but also further challenge the current optimization methods and accelerate the development on new theory and algorithm.

The success of OSB series symposiums motivates us to extend the main themes from optimization & systems biology to computational systems biology and to further foster a wider collaborative platform for biologist, mathematicians, computer scientists, physicians, and engineers. As a result, the conference was formally renamed as International Conference on Computational Systems Biology since 2010. To keep the tradition, the 2010 conference was called as the 4nd International Conference on Computational Systems Biology (ISB2010). In addition to the sponsorships from National Natural Science Foundation of China (NSFC), Academy of Mathematics and Systems Sciences of CAS (AMSS), Shanghai Institutes for Biological Sciences of CAS (SIBS), the conference was further sponsored by Soochow University, Computational Systems Biology Society of ORSC, and Systems Biology Technical Committee of IEEE SMC Society.

We strongly believe that the development of computational methodologies for systems biology is still in its infancy and much research work remains to be done in this area. Computational systems biology holds our common promise to address questions fundamental to our understanding of life and to further lead to practical applications in medicine, drug discovery, and engineering.

## Meeting report

A three-day international conference on Computational and Systems Biology was held in 9-11 September, 2010 (ISB2010) in Suzhou, which is renowned for its beautiful stone bridges, pagodas, and meticulously designed gardens in south China. More than 100 researchers including engineers, physicians, mathematicians, and biologists from China mainland, United States, Germany, Czech Republic, Netherland, Hong Kong, Taiwan, Japan, Korea, Australia, and Singapore enjoyed both academic exchanges and natural scenes.

Following the successful OSB2007 (http://www.aporc.org/ISB/2007/index.php), OSB2008 (http://www.aporc.org/ISB/2008/index.php), and OSB2009 (http://www.aporc.org/ISB/2009/index.php), the purposes of ISB 2010 is to extend the international forum for scientists, researchers, educators, and practitioners to exchange their ideas by presenting research findings and state-of-the-art solutions in this interdisciplinary field, including computational methods and its applications in biosciences and researches on various aspects of Systems Biology.

The Proceedings of the Fourth International Conference on Computational Systems Biology (ISB2010) have been published by World Publishing Corporation (ISBN 978-7-5100- 2407-8/O_820) as Lecture Notes in Operations Research 13 and the proceedings are freely available online (http://www.aporc.org/LNOR/13/). Forty-four papers in this volume cover wide range of computational systems biology and all the papers are indexed by ISTP (Index to Scientific \& Technical Proceedings). Moreover, the reviewers from the Program Committee of ISB2010 selected 16 papers for a special issue in BMC Systems Biology after significant extension of their original versions on the Proceedings. Each submission has been peer reviewed and evaluated by three independent reviewers on the quality, originality, soundness, and significance of its contributions and the significant improvement regarding to the ISB2010 proceeding paper. Here we focus on some of the highlights of the meeting by categorizing and briefly introducing these selected papers.

Roughly, the research in computational systems biology field can be categorized into two classes: methodology development and biological application. The former study is mainly methodology driven: the researchers hold their hammer to find a nail. The latter is mainly biological problem driven: the researchers have their nails and want a proper hammer. The methodology space is unlimited and currently many tools from other fields have been borrowed to solve biological problems. Instead, the research objectives are limited to the basic building blocks of biological science. Realizing this research philosophy of computational systems biology, we classified the 16 selected papers by their research objectives as: sequence, structure, function, and network.

## Analyzing sequence and extending to pathway and networks

Genomic sequence data is the most basic and widely available data with the rapid development of next sequencing technology. Developing sophisticated sequence analysis tools to understand sequence data is the kernel task and will create a significant impact on many aspects of computational systems biology. For instance, sequence alignment method is used to map pathway and regulatory network in this special issue.

Bi-directional gene pairs have received considerable attention for their prevalence in vertebrate genomes. Bingcuan Liu et al. conducted a genome-wide investigation in terms of their sequence composition, functional association and regulatory motif discovery to study the properties of the gene organization and the difference between bi- and uni-directional genes. Chia-Sheng Chuang et al. developed a functional pathway mapping strategy for constructing biological pathways by featuring homologous relationship among various model species. Then they used their computational method to study hypoxia-inducible factors, which are transcription factors that play a crucial role in response to hypoxic stress in living organisms. Rachita Sharma et al. noticed that many regulatory network information has already been determined experimentally for model organisms, but much less has been identified for non-model organisms. Hence, they proposed a method to determine the regulatory links that can be mapped from a model to a non-model organism.

## Predicting protein structure, function, and interactions

Protein structure is important in its role to reveal the molecular bases of how macromolecular complexes and cell networks operate. For instance, characterize the structural features of interacting residues is key to understand protein interaction. To predict protein binding hot spots, Zhenhua Li et al. proposed a new descriptor called “burial level” for characterizing residues, atoms and atomic contacts in protein structures. Then they identified different kinds of deeply buried atomic contacts at different burial levels that are directly broken in alanine substitution. These data then were feeded as input for SVM to predict hot spot residues.

Enzymes are known as the largest class of proteins and their functions are usually annotated by the Enzyme Commission (EC), which uses a hierarchy structure. Yongcui Wang et al. used the protein sequence information to predict enzyme function by considering the hierarchy structure of enzyme protein annotation. Also they carefully extended the existing SVM method to reduce the computational complexity.

Protein-DNA interactions play an important role in many fundamental biological activities such as DNA replication, transcription and repair. JingNa Si et al. present metaDBSite, a meta web server to predict DNA-binding residues for DNA-binding proteins. MetaDBSite integrates the prediction results from six available online web servers: DISIS, DNABindR, BindN, BindN-rf, DP-Bind, and DBS-PRED and it only uses sequence information of proteins.

For protein-protein interaction, Morihiro Hayashida et al. proposed novel methods using conditional random fields for predicting protein-protein interactions by utilizing the fact that mutual information between residues at interacting sites can be higher than that at non-interacting sites.

## Predicting gene function, drug targets, and drug combination

Networks can be utilized to discover novel genes involved in specific biological processes because cellular functions depend on genetic, physical, and other types of interactions. Lin Wang et al. proposed to discover genes involved in the cell cycle process in budding yeast by combining E-MAP data with other high-throughput data, such as gene expression, transcription factor (TF)–DNA binding, and protein phosphorylation.

Shao Li et al. highlighted the importance of multicomponent therapeutics in the control of complex disease. They proposed a “network target”-based paradigm instead of the traditionally single target-based or multiple target-based paradigm for virtual screening and established an algorithm termed NIMS (Network target-based Identification of Multicomponent Synergy) to prioritize synergistic agent combinations. They further applied their NIMS in the empirical multicomponent system traditional Chinese medicine.

For metabolic network, Zhenping Li et al. developed a method based on flux balance analysis of metabolic networks to identify potential drug targets. Their method utilizes two linear programming models to find the steady optimal fluxes of reactions and the mass flows of metabolites in the pathologic state, and then determine the fluxes and mass flows in the medication state with the minimal side effect caused by the medication. Drug targets were identified by comparing the fluxes of reactions in both states and examining the change of reaction fluxes.

## Modelling dynamical biomolecular network

Dynamic modeling is important in metabolic engineering with known metabolic network. For instance, comprehensive kinetic models of microbial metabolism can enhance understanding of system dynamics and regulatory mechanisms, which is helpful in optimizing microbial production of industrial chemicals. Rudong Li et al. developed an improved kinetic model featured with the incorporation of butyryl-phosphate, inclusion of net effects of complex metabolic regulations, and quantification of enzyme activity variations caused by these regulations.

Network dynamics can be used to study the mechanism of complex biological phenomenon. For example, the ability for living cells to respond properly to apoptosis signals is crucial for proper development and maintenance of hemeostasis of multicellular organisms. Chang Gu et al. investigated TNF induced apopotosis, and established a mathematical model without the requirement of bistability. Their simulation revealed a pulse increasing of caspase-3 activation following signalling stimulation to trigger the irreversible death program, which agrees with experimental observations.

Jimmy Omony et al. studied the dynamics of the transcription-translation system for XlnR regulon in Aspergillus niger. Their model was based on Hill regulation functions and used ordinary differential equations. The network response to a trigger of D-xylose is considered and stability analysis was performed. The activating, repressive feedback, and also the combined effect of the two feedbacks on the network behavior were analyzed.

Revealing the multi-equilibrium property of a metabolic network is a fundamental and important topic in systems biology. It is generally difficult to study from both analytical and numerical viewpoint. Hongbo Lei et al. decomposed the network into several building modules and analyzed the SSI metabolic module by using a set of nonlinear ordinary equations with multi-variables. A sufficient and necessary condition was given to describe the injectivity of a class of nonlinear systems, and then, used to study the multi-equilibrium property of SSI modules for a general metabolic network.

## Studying complex disease and stem cell

In addition to dynamic simulation, network is a powerful tool in computational systems biology to probe the fundamental biological problem, such as occurrence and development of complex disease and the recent cell reprogramming. In this special issue, Huarong Zhou et al. noticed the fact that a whole network responsible for a specific phase of diabetes is missing, while a single gene has to be put into a network to evaluate its importance. In the study, they aimed to identify significant transcriptional regulatory networks in the liver contributing to diabetes and performed comprehensive active regulatory network survey in 4 weeks (w), 8-12 w, and 18-20 w Goto-Kakizaki rat liver microarray data by network screening, which is an elegant method to identify the network structure consistent with gene expression data,

Recently human iPS cells (hiPSCs) attract great attention for the application to drug screening and analysis of the mechanisms of diseases. One of the most important biological questions here is how exogenous factors induce changes in the inner and outer cellular states. Shigeru Saito et al. analyzed both RNA profile to reveal gene expression changes and glycan profile to identify structural changes in glycans between four parental somatic cell (SC) lines and nine hiPSC lines that were originally established. They combined standard statistical techniques and a network approach, and showed that there was significant differences in expression between the iPSCs and SCs. Subsequent network analysis of the gene expression and glycan signatures revealed glycan transfer network associated with known epitopes for differentiation based on characteristic changes in the cellular surface states of the hiPSCs.

## Competing interests

The authors declare that they have no competing interests.

## Authors' contributions

YW drafted the manuscript. XSZ and LC read and approved the manuscript.
